# Addressing the undertreatment of mood disorders in Australian youth

**DOI:** 10.1177/00048674241312795

**Published:** 2025-01-22

**Authors:** Jason P Connor, James G Scott, Wayne D Hall, Phong K Thai

**Affiliations:** 1 Discipline of Psychiatry, The University of Queensland, Brisbane, QLD, Australia; 2 National Centre for Youth Substance Use Research, The University of Queensland, Brisbane, QLD, Australia; 3 Child Health Research Centre, The University of Queensland, South Brisbane, QLD, Australia; 4 Queensland Centre for Mental Health Research, Wacol, QLD, Australia; 5 Child and Youth Mental Health Service, Children’s Health Queensland, South Brisbane, QLD, Australia; 6 Queensland Alliance for Environmental Health Sciences, The University of Queensland, Brisbane, QLD, Australia

**Figure 1.**
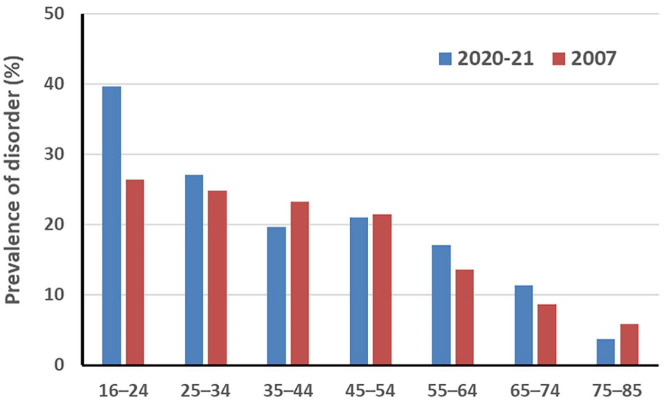
Prevalence of any 12-month mental health disorder in Australia, by age (data from the National Study of Mental Health and Wellbeing).

Compared to other Australians, youth are disproportionately affected by mental illness and depression is a key driver of morbidity and mortality in this age group. In 2020/2021, the National Study of Mental Health and Wellbeing reported almost 40% of 16- to 24-year-olds had a mental disorder in the previous year (Figure 1). Of concern, only half of Australian adolescents with mental health problems sought treatment (Islam et al., 2022).

Barriers to mental health care in Australian youth include poor access to a regular GP, challenges accessing providers, low levels of health literacy and rurality. Young people with depression may also experience stigma and discrimination, which impede help-seeking. Their lack of health literacy about depression treatment and stigma from self and others is often compounded by parents’ and caregivers’ lack of understanding and cultural awareness of mental health disorders and their treatment.

Cost-effective non-pharmaceutical approaches such as brief behavioural interventions and online self-help tools can be effective in persons with lower severity of depression. Royal Australian and New Zealand College of Psychiatrists clinical practice guidelines recommend behavioural activation and psychological interventions as first line management of mood disorders. But accessing those interventions can be expensive, particularly so for young Australians. Australia’s Better Access initiative provides Medicare-rebated services from psychologists, social workers and occupational therapists who provide evidence-based behavioural treatments for depression, as part of a mental health care plan initiated by a general practitioner. Considerable ‘gap’ fees and waitlist times are barriers to treatment. Almost 70% of people who receive psychology treatments pay an out-of-pocket median gap fee of $90AUD. Lack of affordability and poor availability of mental health professionals often means that psychological support for mental disorders is not available for many young Australians.

**Figure 2.**
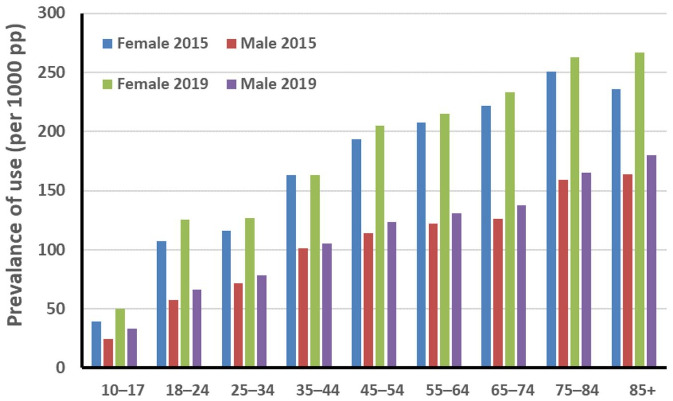
Antidepressant use in Australia by age (data from de Oliveira Costa et al. (2023)).

For young people with moderate to severe and severe mood disorder, available effective treatments are more limited, leading to a low proportion of youth with depression receiving treatment. Australian and New Zealand clinical practice guidelines recommend treating moderate to severe depression with psychological interventions and lifestyle medicine in combination with antidepressant medication. Yet, antidepressant use is lowest in the 10- to 24-year age group despite the very high prevalence of mental illness in this demographic (Figure 2) (de Oliveira Costa et al., 2023).

There are several possible explanations for the low level of antidepressant medication use by young Australians. There are restricted pharmacological options for this age group. In Australia, fluoxetine is the only antidepressant medication recommended for patients under 18 years. In an Australian double-blind, multi-centre, placebo-controlled trial, there was no evidence that the addition of fluoxetine to cognitive behavioural therapy in young people with moderate-to-severe depression further reduced depressive symptoms compared to placebo (Davey et al., 2019).

Low rates of antidepressant prescriptions to young people could also reflect concern about the possibility that antidepressants increase suicidality in young people. Black box warnings about suicidality may have contributed to the under-use of antidepressant medications in young people. A recent meta-review (Boaden et al., 2020) concluded that there was a minimal risk of increased suicidality with antidepressants in young people. Valid and reliable open-access multi-item suicide risk assessments are available and can be incorporated in clinical reviews.

Significant reforms to the mental health system are required if it is to better meet youth mental health needs. An increased focus on prevention is urgently needed. A universal public health emphasis aiming to increase physical activity and improve sleep and nutrition and social relations would improve mental and physical health (Rice et al., 2014; Sampasa-Kanyinga et al., 2020). An accessible example of online interventions to increase social engagement is Australia’s Orygen Virtual Worlds, an avatar-driven virtual world where young people can connect and interact. However, online programmes and ‘apps’ supported by research demonstrating their effectiveness are less common. For example, a 2019 study of mental health apps found that while almost two-thirds (64%) made positive claims of effectiveness, only 2.7% reported direct research evidence to support these claims (Larsen et al., 2019). Those that do meet the *Australian National Safety and Quality Digital Mental Health Standards* may not be accessible after the research study concludes, or there is a fee-for-service model applied to some or all of the online programme content. Some examples of fully open access programs include MOST, MindSpot and Headspace.

Education, reassurance and online support may be sufficient for the large number of young Australians with mild symptoms of depression and anxiety. Leveraging young people’s engagement in social media as a preferred mode of communication and emotional support may offer additional management options. These broader strategies would increase workforce capacity to provide mental health care to youth with more severe and persistent illness. Mental health service reform must be done in combination with programmes to reduce stigma, such as the forthcoming *National Stigma and Discrimination Reduction Strategy*.

Access to appropriate treatment may be compounded by a discordance between the mental health needs of youth and the types of services that are provided. While young people with mild depression and anxiety should be supported by the strategies mentioned above, those with more distress and impairment should be able to access psychiatrists and clinical psychologists. For young people with the most severe illnesses, access to high-quality public mental health care is critical. However, there has been an underinvestment in these services over many years leading to their inability to meet community demand. Coordination and integration of care is needed across the mental health continuum.

Why has there been such a significant increase in common mental disorders among youth over the past 15 years? While there has been considerable public investment in services, such as Headspace, the root cause of the youth mental health crisis in Australia and other countries in the Global North remains an enigma. A comprehensive explanation of why so many young Australians now live with common mental disorders is urgently needed. Prevention is key; the provision of services alone will not address this health crisis. There is a temptation to blame social media because their use by young people has often been associated with higher levels of anxiety, body image dissatisfaction and eating problems. Separating cause and effect is a challenge. We think it is more likely that there are a multitude of factors each exerting a small effect, which are causing the decline in the mental health of youth in high-income countries across the globe. Social media may amplify the effects of these factors.

Comprehensive public health initiatives that positively influence youth well-being and reduce symptoms of common mental disorders need to be combined with improved coordination and integration of mental health services in order to effectively improve the mental health of Australian youth.
